# Protocol for a randomized, placebo-controlled, double-blind phase IIa study of the safety, tolerability, and symptomatic efficacy of the ROCK-inhibitor Fasudil in patients with Parkinson’s disease (ROCK-PD)

**DOI:** 10.3389/fnagi.2024.1308577

**Published:** 2024-02-14

**Authors:** Andreas W. Wolff, Helen Bidner, Yvonne Remane, Janine Zimmer, Dag Aarsland, Olivier Rascol, Richard K. Wyse, Alexander Hapfelmeier, Paul Lingor

**Affiliations:** ^1^Department of Neurology, Klinikum Rechts der Isar, School of Medicine, Technical University of Munich, Munich, Germany; ^2^Münchner Studienzentrum (MSZ), School of Medicine, Technical University of Munich, Munich, Germany; ^3^Department of Clinical Pharmacy and Drug Safety Center, Leipzig University, Leipzig, Germany; ^4^Department of Old Age Psychiatry, Institute of Psychiatry, Psychology and Neuroscience, King’s College London, London, United Kingdom; ^5^Centre for Age-Related Research, Stavanger University Hospital, Stavanger, Norway; ^6^Clinical Investigation Center CIC1436, Departments of Clinical Pharmacology and Neurosciences, University of Toulouse 3, University Hospital of Toulouse, INSERM, Toulouse, France; ^7^Cure Parkinsons, London, United Kingdom; ^8^Institute of AI and Informatics in Medicine, School of Medicine, Technical University of Munich, Munich, Germany; ^9^Institute of General Practice and Health Services Research, School of Medicine, Technical University of Munich, Munich, Germany; ^10^German Center for Neurodegenerative Diseases (DZNE), Munich, Germany; ^11^Munich Cluster of Systems Neurology (SyNergy), Munich, Germany

**Keywords:** Parkinson’s disease, clinical trial, safety, tolerability, symptomatic efficacy, disease modification, Fasudil, ROCK inhibition

## Abstract

**Background:**

The Rho-kinase (ROCK) inhibitor Fasudil has shown symptomatic and disease-modifying effects in Parkinson’s disease (PD) models *in vitro* and *in vivo*. In Japan, Fasudil has been approved for the treatment of subarachnoid haemorrhage since 1995 and shows a favourable safety profile.

**Objectives/design:**

To investigate the safety, tolerability, and symptomatic efficacy of ROCK-inhibitor Fasudil in comparison to placebo in a randomized, national, multicenter, double-blind phase IIa study in patients with PD.

**Methods/analysis:**

We plan to include 75 patients with at least ‘probable’ PD (MDS criteria), Hoehn and Yahr stages 1–3, and age 30–80 years in 13 German study sites. Patients must be non-fluctuating and their response to PD medication must have been stable for 6 weeks. Patients will be randomly allocated to treatment with the oral investigational medicinal product (IMP) containing either Fasudil in two dosages, or placebo, for a total of 22 days. As primary analysis, non-inferiority of low/high dose of Fasudil on the combined endpoint consisting of occurrence of intolerance and/or treatment-related serious adverse events (SAEs) over 22 days will be assessed in a sequential order, starting with the lower dose. Secondary endpoints will include tolerability alone over 22 days and occurrence of treatment-related SAEs (SARs) over 22 and 50 days and will be compared on group level. Additional secondary endpoints include efficacy on motor and non-motor symptoms, measured on established scales, and will be assessed at several timepoints. Biomaterial will be collected to determine pharmacokinetics of Fasudil and its active metabolite, and to evaluate biomarkers of neurodegeneration.

**Ethics/registration/discussion:**

After positive evaluation by the competent authority and the ethics committee, patient recruitment started in the 3rd quarter of 2023. ROCK-PD is registered with Eudra-CT (2021-003879-34) and clinicaltrials.gov (NCT05931575). Results of this trial can pave way for conducting extended-duration studies assessing both symptomatic efficacy and disease-modifying properties of Fasudil.

## 1 Introduction

Parkinson’s disease (PD) is a progressive neurodegenerative disorder with a multi-factorial pathogenesis. Multiple molecular pathways, such as dysfunction in the ubiquitin-proteasome system, defective lysosomal degradation and autophagy, protein aggregation, mitochondrial malfunction, and oxidative stress, have been implicated as disease mechanisms (Obeso et al., 2017; Burré et al., 2018). Up to now, only symptomatic treatments are available and there is a great need to develop disease-modifying therapies (Stocchi and Olanow, 2013). Because of the multifactorial pathogenesis of PD, disease-modifying treatments will also have to address multiple mechanisms. Fasudil is a small-molecule inhibitor of Rho-associated coiled coil kinase (ROCK), an enzyme that is known to mediate vasoconstriction and vascular remodelling by regulation of the actin cytoskeleton. Interestingly, inhibition of ROCK was also demonstrated to increase actin fluidity and thus regenerative capacity in neuronal growth cones, to activate cell survival and protein synthesis pathways (via Akt, PTEN, and mTOR) (Li et al., 2005; Park et al., 2011), as well as to modulate microglial function by changing the microglial phenotype from the rather cytotoxic M1 to the rather cytoprotective M2 (Zhang et al., 2013). ROCK expression is increased in several neurodegenerative disorders, including Amyotrophic lateral sclerosis (ALS), Alzheimer’s disease, and PD (Koch et al., 2018). The ROCK-inhibitor Fasudil was originally developed as a vasodilatory drug and was licensed in Japan in 1995 for the treatment of vasospasms following subarachnoid haemorrhage (SAH). It has been tested in numerous clinical trials since, most frequently for cardiovascular diseases (Ruan et al., 2019; Hou et al., 2022). Fasudil is primarily metabolized in the liver, resulting in the formation of its active metabolite, Hydroxyfasudil, subsequently undergoing renal excretion. Clinical meaningful quantities of Hydroxyfasudil also cross the blood–brain barrier (Taniguchi et al., 2014). This makes Fasudil a potential candidate for the treatment of central nervous system disorders. The effects of Fasudil were assessed in a phase 3 trial in patients with acute ischemic stroke, where Fasudil treatment significantly improved clinical outcome (Shibuya et al., 2005). Additionally, beneficial effects on survival and motor function have been observed in animal models of ALS (Takata et al., 2013; Tönges et al., 2014; Günther et al., 2017) and other neurodegenerative disorders, e.g., Alzheimer’s disease (Koch et al., 2018). Currently, an investigator-initiated, international multicenter trial is ongoing to evaluate safety, tolerability, and efficacy of Fasudil in patients with ALS (ROCK-ALS, NCT03792490, Eudra-CT: 2017-003676-31)^1^ (Lingor et al., 2019). We and others demonstrated that Fasudil exerts neuroprotective and pro-regenerative effects in PD models *in vitro* and *in vivo*: Fasudil protected dopaminergic tyrosine hydroxylase-positive nigral neurons from degeneration, increased their regeneration and resulted in increased striatal dopamine levels accompanied by a behavioural improvement in rodents (Tönges et al., 2012; He et al., 2016; Yang et al., 2020). Importantly, Fasudil specifically binds to alpha-synuclein at two tyrosine residues (Y133 and Y136) and attenuates aggregation of alpha-synuclein *in vitro* and *in vivo*, independently from its effects on the actin cytoskeleton (Tatenhorst et al., 2016). Furthermore, Fasudil has shown symptomatic efficacy by dopaminergic effects (Sortwell et al., 2013) and reduction of L-Dopa-induced dyskinesia (Lopez-Lopez et al., 2020) in preclinical rodent models, suggesting that it could also be beneficial for patients in the short term. Taken together, Fasudil has shown multiple disease-modifying properties in models of PD *in vitro* and *in vivo* making it a highly promising candidate for disease-modifying therapeutic trials in PD (Tönges et al., 2012; Tatenhorst et al., 2014, 2016; Koch et al., 2018). An intravenous formulation of Fasudil is licensed in Japan, oral (tablet) formulations, including extended release formulations, were previously used in clinical trials in humans (Vicari et al., 2005; Fukumoto et al., 2013) and a good enteric bioavailability of the drug has been documented (Hinderling et al., 2007). The longest published exposure to Fasudil in humans was 8 and 12 weeks (angina pectoris and pulmonary arterial hypertension) (Vicari et al., 2005; Fukumoto et al., 2013). Side effects included allergic skin reactions, a mild drop in systolic blood pressure, and reversible renal impairment without major safety concerns. In view of the ample preexisting clinical experience with Fasudil and its well-known safety profile (Suzuki et al., 2007, 2008) it represents an excellent candidate for repositioning as a disease-modifying therapy in PD (Koch et al., 2018).

We present here the design of a randomized, placebo-controlled, national, multicenter, double-blind phase IIa study for safety, tolerability, and symptomatic efficacy of the ROCK-inhibitor Fasudil in patients with PD. So far, ROCK-inhibition has not yet been evaluated in the treatment of PD making this trial unique. Considering the potential symptomatic and disease-modifying effects of Fasudil in PD, patients could benefit from attenuation of disease-progression as well as from immediate clinical improvement.

## 2 Methods and analysis

### 2.1 Objectives and design

**Figure 1:**
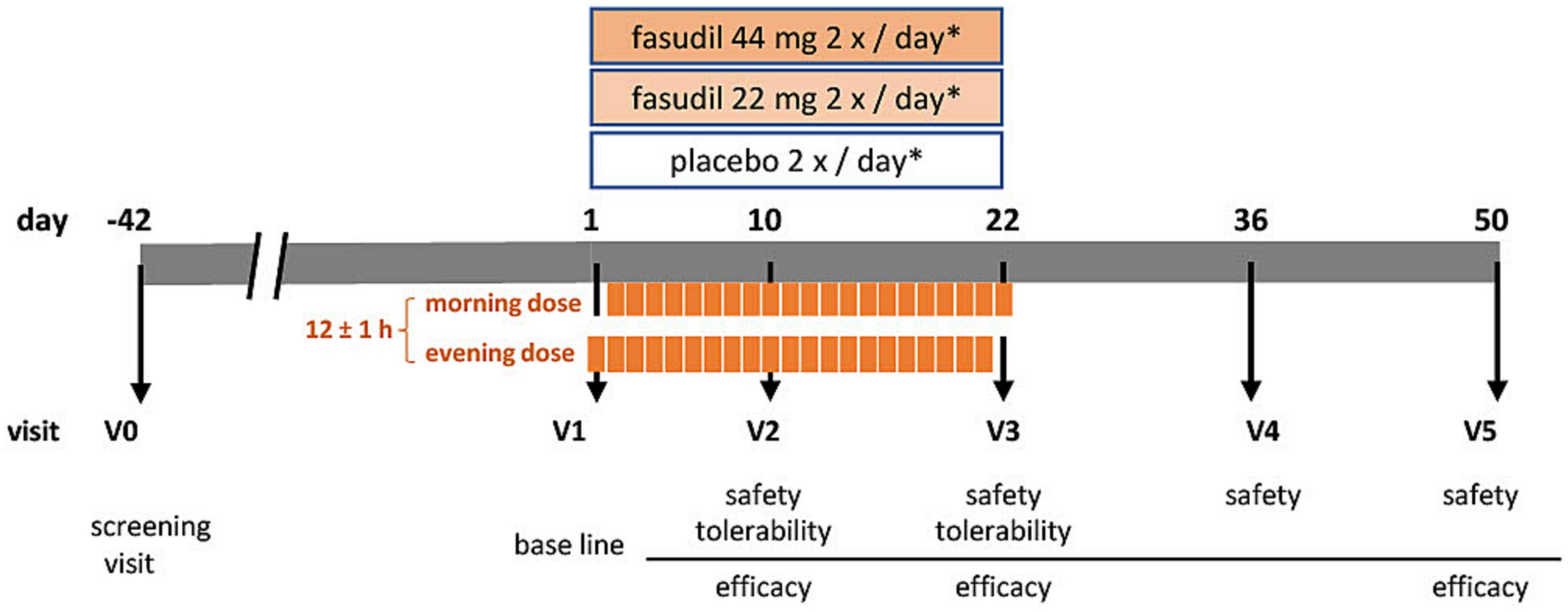
Visit schedule of the ROCK-PD study *IMP (investigational medicinal product either containing Fasudil [44 mg or 22 mg] or placebo) administration at day 1 and day 22 once daily, on days 2–21 twice daily.

The objective of the ROCK-PD trial is to establish a safety and tolerability profile of the ROCK-inhibitor Fasudil by testing for non-inferiority against placebo, and asses symptomatic efficacy in patients with PD. It is designed as a multi-center, prospective, interventional, randomized, double-blind, placebo-controlled, parallel-group, phase IIa trial. In brief, patients with PD, who are non-fluctuating on their antiparkinsonian medication, will be recruited to receive either Fasudil in two dosages, or placebo, over a period of 22 days. Two follow-up visits (until day 50) will establish safety and symptomatic effects during wash-out (Figure 1). The SPIRIT guidelines were followed in the preparation of the trial protocol (Chan et al., 2013; Supplementary Table S1) and study results will be published in accordance with the CONSORT guidelines (Moher et al., 2010).

### 2.2 Participants

#### 2.2.1 Inclusion and exclusion criteria

Patients with a diagnosis of at least ‘probable’ PD according to MDS criteria (Postuma et al., 2015), and a Hoehn and Yahr stage 1–3, aged 30 to 80 years are eligible for participation in the ROCK-PD trial. Furthermore, patients must be non-fluctuating (no wearing-off, no dyskinesia) and stable on symptomatic PD medication for at least 6 weeks before screening and baseline. Atypical, secondary parkinsonian syndromes, or any other medical condition known to have an association with parkinsonian syndromes, which might confound or obscure the diagnosis of PD are excluded. Other main exclusion criteria are a history of intracranial bleeding, known intracerebral aneurysms, or Moyamoya disease (due to the Fasudil label). In case of a positive family history for the aforementioned conditions, MR- or X-ray-based imaging not older than 24 months must confirm their absence. Patients with drug-treated pulmonary hypertension, drug-treated arterial hypotension, or unstable or uncontrollable arterial hypertension are also excluded from the trial. Conditions interfering with the safety measurements of this trial, such as confirmed hepatic or renal insufficiency are additional exclusion criteria. The full list of inclusion and exclusion criteria is provided in Table 1.

**Table 1:** Inclusion and exclusion criteria of the ROCK-PD trial.

*Inclusion criteria*
Patients with a diagnosis of at least ‘probable’ PD according to MDS criteria (Postuma et al., 2015). Hoehn and Yahr stage 1–3. must be non-fluctuating (no wearing-off, no dyskinesia) and stable on symptomatic PD medication for at least 6 weeks. Age: 30–80 years. Women of childbearing age must be non-lactating and surgically sterile or using a highly effective method of birth control and have a negative pregnancy test. Acceptable methods of birth control with a low failure rate (i.e., <1% per year) when used consistently and correct are for example implants, injectables, combined oral contraceptives, hormonal intrauterine devices (IUDs), sexual abstinence, or vasectomized partner. Capable of thoroughly understanding all information given and giving full informed consent according to GCP.
*Exclusion criteria*
Atypical, secondary Parkinsonian syndromes, PD mimics, or any other medical condition known to have an association with Parkinsonian syndromes, which might confound or obscure the diagnosis of PD. Patients with a history of intracranial bleeding, known intracerebral aneurysms or Moyamoya disease, or positive family history for the above. If family history positive, MR- or x-ray-based cranial imaging not older than 24 months must confirm absence of bleeding, aneurysms, or Moyamoya disease. Presence of any concomitant life-threatening disease or impairment likely to interfere with functional assessment. Patients with known arterial hypotension (resting blood pressure <90/60 mmHg), previous hypotensive episodes, or requiring treatment, such as fludrocortisone, midodrine, etilefrine, cafedrine, or theodrenaline. Patients with an uncontrollable or unstable arterial hypertension (resting blood pressure >180 mmHg systolic and/or >120 mmHg diastolic, whether or not under current antihypertensive medication). Known pulmonary hypertension and any medication prescribed for treatment of pulmonary hypertension. Confirmed hepatic insufficiency or abnormal liver function (stable ASAT and/or ALAT >3 times the upper limit of the normal range) and determined to be non-transient through repeat testing. Renal insufficiency with a glomerular filtration rate (GFR) <60 mL/min/1,73 m^2^ (calculated by MDRD equation) and determined to be non-transient through repeat testing. Major psychiatric disorder, significant cognitive impairment or clinically evident dementia precluding evaluation of symptoms. Hypersensitivity to any component of the IMP. Liable to be not cooperative or comply with the trial requirements (as assessed by the investigator), or unable to be reached in the case of emergency. Pregnant or breast-feeding females or females with childbearing potential, if no adequate contraceptive measures are used. Previous participation in another clinical study involving trial medication within the preceding 12 weeks or five terminal half times of the longest to be eliminated trial medication (whichever is longer) or previous participation in this trial.

#### 2.2.2 Recruitment

Patients will be recruited and assessed for eligibility at currently 13 study sites in Germany. All study sites are part of the German competence network for Parkinson’s disease (accessed on March 15, 2023)^2^ and are actively involved in the treatment of several hundred PD patients per year. A web site has been established to inform referring centers about the trial (accessed on September 27, 2023).^3^ All patient assessments including the screening visit will be performed at the study sites. Recruitment started in the 3rd quarter 2023 and is anticipated to be completed by the 3rd quarter 2024. Throughout the clinical trial, recruitment rates will be monitored for each study site.

### 2.3 Randomization and blinding

Participants will be randomized into three parallel groups at an allocation ratio of 1:1:1 (Figure 1). There will be two Fasudil treatment arms (low and high dosage) and one placebo arm. Allocation will be performed based on a computer-generated randomization list. All procedures will be performed double-blind (patient- and investigator blind). During the clinical trial, unblinding may only occur in the case of an emergency, where the choice of treatment depends on the study subject’s therapy assignment. Emergency envelopes for the unblinding of study participants are provided.

### 2.4 Drug procurement and intervention

Fasudil hydrochloride hydrate (Eril 30 mg, 15 mg/mL ampoules) has been imported from Asahi Kasei Pharma Corporation, Tokyo, Japan, by a licensed importer and provided to the central pharmacy for this clinical trial (Pharmacy at University of Leipzig Medical Center, Germany), where it will be decanted and blinded as IMP (22 or 44 mg, 15 mg/mL stock solution). Due to the bitter taste of Fasudil, the placebo formulation requires the addition of a bittering agent. As a bittering agent, Quinine dihydrochloride solution (Quinina Labesfal 500 mg, 250 mg/mL ampoules) has been imported from Labesfal – Laboratórios Almiro, Santiago de Besteiros, Portugal, by a licensed importer and provided to the central pharmacy where it will be re-packaged as IMP (12.5 mg, 250 mg/mL stock solution). The IMP will be produced, packaged, stored, blinded, labelled, and shipped to the trial sites in accordance with the legal requirements and good manufacturing practice. Participants will orally administer the IMP every 12 h, with a tolerance window of ±1 h. Before oral self-administration, participants will supplement the IMP with 30 mL Glucose 40% solution.

### 2.5 Study procedures

A total of six visits (V0-V5) will be performed (see Table 2). Informed consent will be obtained before any study-specific procedure. During the screening visit (V0), eligibility according to inclusion and exclusion criteria, medical history, vital signs, concomitant medication, height, and weight will be established. A physical examination will be complemented by assessment of modified Hoehn and Yahr scale (mHY) and laboratory tests for safety parameters. This visit may take place up to 42 days in advance to the start of the treatment period. At the beginning of the treatment period, a baseline visit will be performed (V1). At this stage, we will reconfirm the inclusion and exclusion criteria, assess baseline measurements of laboratory parameters, vital safety indicators, and symptom-related assessment scales. These scales include the MDS-Unified PD Rating Scale I-IV (MDS-UPDRS), PD Quality of Life Scale (PDQ-8), PD Non-Motor Symptom Questionnaire (NMSQuest), Montreal Cognitive Assessment (MoCA), Beck’s Depression Inventory-II (BDI-II), and Global Impression of Improvement score (CGI-I [clinician], PGI-I [patient]). Participants will start intake of IMP in the evening after V1 and continue for 22 days. On the morning of day 22 the last dose should be administered at the study site. Safety parameters (laboratory measures and vital signs) will be assessed at all study related visits. These are on day 10 (±2 days; V2) and day 22 (−2 days; V3), during the treatment period, as well as day 36 (±3 days; V4) and 50 (±3 days; V5) during the washout period. At visits V2 and V3, tolerability will be recorded. Symptomatic efficacy will be assessed during treatment period (V2: subset of scales: MDS-UPDRS, subscales III and IV, NMSQuest, PGI-I, CGI-I), on the last day of treatment (V3), and after the wash-out phase (V5). We will collect biomaterials, including blood, cerebrospinal fluid (CSF), and tear fluid, for the purpose of establishing the pharmacokinetics of oral Fasudil and its active metabolite, Hydroxyfasudil. Additionally, these samples will be used to measure target engagement (ROCK activity) and for biobanking.

**Table 2:** Trial visit schedule.

Visit item	V0 screening	V1 BL	V2	V3	V4	V5
Day (d)	0	1	10	22	36	50
Permitted delta in days (±) to V1	−42	0	±2	−2	±3	±3
IMP orally 2x/d, on day 1 and day 22 1x/d		x	x	x		
*Screening assessment/randomization*						
Patient information and informed consent	X					
Inclusion/exclusion criteria	X	X				
Demographics	X					
Medical history	X					
Diagnosis according to MDS criteria	X					
Modified Hoehn and Yahr scale (mHY)	X					
Physical examination	X					
Randomization	X^*^					
*Recurrent additional status data*						
Concomitant treatment history	X	X	X	X	X	X
Vital signs (pulse, BP)	X	X	X	X	X	X
Height (only at screening), weight	X	X		X		X
*Endpoint assessment*						
Adverse events/tolerability (acquisition only after first drug intake)		X	X	X	X	X
MDS-UPDRS, subscales I and II^**^		X		X		X
MDS-UPDRS, subscales III and IV^**^		X	X	X		X
NMSQuest		X	X	X		X
MoCA^***^		X		X		X
BDI-II		X		X		X
PDQ-8		X		X		X
PGI-I (patient) and CGI-I (health professional)			X	X		X
*Laboratory assessments*						
Safety parameters^****^	X	X	X	X^§^	X	X
Pregnancy test (if female and fertile)	X	X	X	X	X	X
*Samples for exploratory analyses and biobanking*						
Fasudil/Hydroxyfasudil in CSF and plasma		P		C^#^&P^#^		P
ROCK activity in plasma		X		X		X
*Additional samples for biobanking (separate ICF)*						
Serum/plasma/tear fluid		X		X		X
EDTA blood		X				
CSF				X		

^*^Randomization takes place between V0 and V1 by central pharmacy. ^**^MDS-UPDRS assessment has to be performed at the same interval to IMP intake in each patient (±1 h). ^***^Different versions of MoCA will be used at each assessment. ^****^RBC, WBC, PLT, creatinine, GFR (MDRD), ALAT, ASAT, GGT, and CK. ^§^Additional parameters: INR, PTT. ^#^Sample collection has to be performed 120 ± 60 min after the intake of last trial medication.

#### 2.5.1 Harmonisation of cohorts and methods

ROCK-PD exclusively enlists academic hospitals known for their expertise in conducting PD clinical trials. Clinical assessments employ established scales to evaluate PD features, administered by trained personnel at all participating centers. Although training certificates are not mandatory, the consistent expertise of these teams ensures the accuracy of clinical evaluations. Conducting the laboratory work-up is entrusted to trained personnel following rigorous protocols. A detailed biomaterial protocol has been prepared, providing a transparent account of the processes involved in handling and analysing biomaterials. Oversight is maintained by a monitoring board, conducting regular virtual and on-site visits. This proactive monitoring ensures protocol adherence and data integrity, instilling confidence in the reliability of the collected data.

### 2.6 Outcomes

#### 2.6.1 Primary outcome

The composite primary endpoint consists of the occurrence of intolerance, defined as termination of treatment because of treatment-related adverse effects (AE), and/or self-reported or pre-defined (laboratory, vital signs) treatment-related serious adverse effects (SAE) from day 1 (V1) to day 22 (V3) of the treatment period.

#### 2.6.2 Secondary outcomes

The occurrence of intolerance (termination of treatment because of treatment-related AE; time frame: from V1 to V3) and the occurrence of self-reported and pre-defined (laboratory, vital signs) treatment-related SAEs (from V1 to V3 and V1 to V5) will be assessed alone as two separate secondary outcomes. Furthermore, the symptomatic efficacy of oral Fasudil will be assessed as change of MDS-UPDRS (part I to IV from V1 to V3 and V1 to V5; part I and II from V1 to V3 and V1 to V5; part III and IV from V1 to V2, V1 to V3, and V1 to V5), the change of PDQ-8 (from V1 to V3 and V1 to V5), the change of NMSQuest (from V1 to V2, V1 to V3, and V1 to V5), the change of MoCA (from V1 to V3 and V1 to V5), the change of BDI-II (from V1 to V3 and V1 to V5), and the CGI-I/PGI-I (at V2, V3, and V5).

#### 2.6.3 Exploratory endpoints and biomaterial collection

Biomaterial, consisting of blood (EDTA-blood, plasma, serum, and peripheral blood mononuclear cells), tear fluid (collected with Schirmer test strips), and cerebrospinal fluid (CSF), will be collected at different timepoints throughout the study (Table 2). Pharmacokinetics of oral Fasudil and its active metabolite Hydroxyfasudil in patients with PD will be established by measurements in plasma (V1, V3, V5) and CSF (V3). ROCK activity will be quantified in the plasma at three timepoints, once before treatment (V1), once during treatment (V3), and once at the end of the wash-out period (V5). Additionally, biological samples will be available for exploratory analyses in future biomarker studies. Except for safety assessments, participation in the biomaterial collection is not a mandatory part of the ROCK-PD trial. All biomaterial samples not deemed for immediate analysis will be transferred and stored at the biobank of the Department of Neurology, School of Medicine, Technical University of Munich, Munich, Germany.

### 2.7 Safety

The clinical trial will be monitored by an independent Safety Monitoring Board (SMB). The SMB will conduct regular safety reviews throughout the clinical trial to assess the progress of the trial. All adverse events (AE) will be collected from V1 (start of IMP intake). Each AE is to be classified by the investigator as “serious” or “non-serious.” This classification of the gravity of the event determines the reporting procedures to be followed. The intensity of AE will be graded using the Common Terminology Criteria for Adverse Events of the U.S. Department of Health and Human Services. All SAE will be subject to a second assessment by a medical expert, who will be independent from the sponsor. The second assessor will monitor for a relationship between SAE and IMP and expectedness of SAE (derived from reference safety information). All serious adverse reactions (SAR) will be determined a suspected unexpected serious adverse reaction (SUSAR) and require expedited reporting according to the applicable legal framework (competent authorities, ethic committees, clinical trial sites).

### 2.8 Data recording and study monitoring

The documentation of the clinical trial data in adherence with the GCP-guidelines and the trial protocol is the responsibility of the investigator at the individual trial site. All essential documents will be kept in the Investigator Site File (ISF), which will be stored at the trial site in accordance with ICH GCP. Original data (source documents) remain in hospital medical records and information in the eCRF must be traceable and consistent with the original data. Source documents are, e.g., laboratory results, measurements of clinical scales, vital sign measurements, and questionnaires. No information in source documents about the identity of the patients will be disclosed. Data collected in this clinical trial and except data for exploratory analysis must be entered in an eCRF, which has to be completed by the investigator or authorized trial personnel and signed by the investigator. This also applies for those patients who do not complete the clinical trial. Every case of premature termination must be evaluated by the investigator, reasons for the termination assessed, and documented in the eCRF. The principal investigator holds the responsibility for ensuring the accuracy, completeness, and punctuality of all data submitted to the sponsor in the eCRFs and in all mandatory reports.

After database lock, the principal investigator will receive the study site data for archiving. Data are administered and processed by data management of the Münchner Studienzentrum (MSZ) with the support of a study database (eCRF). The evaluation of the data takes place by programmed validity and consistency checks.

Monitoring activities are performed to ensure that the clinical trial is conducted in accordance with the trial protocol, the principles of GCP, and local legislation. A monitoring plan will be applied, describing the scope of the monitoring activities in detail. A monitoring visit report is prepared for each visit describing the progress of the clinical trial and all identified problems. If a pandemic situation will not allow on-site monitoring, then remote monitoring or monitoring by phone is possible if data protection aspects are considered.

### 2.9 Sample size calculation

Expected probability for occurrence of the composite endpoint in the placebo arm is 5% at end of clinical trial. The two Fasudil arms are expected to reach about 12% each (according to the summary of product characteristics [SmPC] of Eril). A non-inferiority margin of 25% for the difference between Fasudil and placebo will be used based on data from previously published trials showing rates of discontinuation and severe adverse events of up to ~25% in patients treated with drugs that are in current clinical use for PD. For example, increased mortality was observed under selegiline/levodopa treatment (Lees, 1995), while side-effects (e.g., somnolence, dizziness, nausea) resulted in discontinuation in pramipexole-treated patients (Nissen et al., 2012; Schapira et al., 2013; Wang et al., 2017). Both drugs are in current use for PD. We thus consider a non-inferiority margin of ~25% as clinically and ethically acceptable for a drug that can have potentially disease-modifying properties and symptomatic effects. The two Fasudil arms will be tested against placebo in a hierarchical order, starting with the lower dose. This sequential procedure does not necessitate adjustment of the alpha level for multiple testing (Committee for Proprietary Medicinal Products (CPMP), 2002). According to the procedure no confirmatory claim can be based on the second test if the first test did not reach statistical significance. The significance levels are therefore each set to 10%, resulting to an overall type-I error of 10%. This error level was chosen in concordance with the recommendation of the European Medicines Agency (Committee for Medicinal Products for Human Use (CHMP), 2005). Given the aforementioned expected event rates, the non-inferiority margin and the alpha level of 10%, the sample size in each group needs to be 22 for a one-sided two-group test of proportions to reach a power of 80% to reject the null hypothesis that Fasudil is inferior to placebo in terms of its composite primary endpoint (calculated by nQuery Advisor V7.0, Statcon GmbH, Witzenhausen, Germany). A total of 3 × 25 = 75 patients are included in order to account for possible dropouts (for reasons other than intolerance; 12% drop-out).

### 2.10 Statistical analysis

#### 2.10.1 Analysis set

The primary analysis will be performed on the safety analysis set, consisting of all patients randomized into the clinical trial and who took at least one dose of study medication, allocated to the treatment actually received (analysis “as treated,” not “as randomized”), as the primary analysis concentrates on safety and/or tolerability. Patients with missing primary endpoint assessments will be excluded from this analysis. The full analysis set (FAS) consists of all patients randomized into the clinical trial who took at least one dose of study medication and will be analyzed according to the intention-to-treat (ITT) principle, i.e., “as randomized.” If all patients receive the treatment for which they were randomized, the safety analysis set and the FAS are identical. The per-protocol set (PPS) consists of all patients in the FAS who have no major protocol deviations. Patients with protocol deviations will be identified in a blinded manner prior to database lock. Safety and/or tolerability endpoints will be analyzed on the safety set. Analysis of the primary endpoint will additionally be performed on the PPS, as it is more conservative in the non-inferiority setting. All other endpoints will be analyzed on the FAS and PPS. Missing values will not be imputed in this clinical trial.

#### 2.10.2 Primary endpoint analysis

The overall confirmatory significance level is set at 10%. The two primary hypotheses are that each Fasudil arm is non-inferior to the placebo arm referring to the composite primary endpoint. The two hypotheses are tested in a sequential order, first comparing arm B (=lower dose) against placebo and second comparing arm A (=higher dose) against placebo. A confirmatory claim can only be based on the second test if the first test did reach statistical significance. For each of the primary hypothesis tests, an exact one-sided 90% confidence interval (CI) of the differences (defined as placebo minus Fasudil) in the primary outcome will be computed to test non-inferiority. Statistical significance of non-inferiority is reached if the lower limit of the respective 90% CI is above the non-inferiority margin of −25%. Patients who leave the clinical trial prematurely due to reasons other than drug intolerance and treatment-related SAEs (e.g., refusal to come to follow-up visits, relocation to another city), will be excluded from the primary endpoint analysis.

#### 2.10.3 Secondary endpoint analysis

Exact Mann–Whitney U-tests will be used for hypothesis testing of group differences in the ordinal and continuous secondary endpoints using two-sided exploratory significance levels of 5%. *T*-tests will be used in case of normal distribution. No adjustment for multiple comparisons will be made due to the exploratory nature of these analyses. Descriptive statistics will be computed for each endpoint by the treatment arm.

## 3 Discussion

### 3.1 Rationale for the choice of Fasudil

Fasudil, a ROCK-inhibitor, has been developed as a vasodilatory drug and is licensed for the treatment of vasospasms after intracranial hemorrhage. There is strong preclinical evidence that Fasudil can be beneficial for different neurodegenerative diseases. In preclinical models of PD, ROCK-inhibition by Fasudil displays promising properties to counteract PD-related pathology on different levels: Fasudil was shown to (1) counteract alpha-synuclein aggregation, a central neuropathological hallmark of PD (He et al., 2016; Tatenhorst et al., 2016), (2) enhance dopaminergic survival in the substantia nigra and maintain striatal dopaminergic terminals, the neuroanatomical basis of motor dysfunction in PD (Tönges et al., 2012; He et al., 2016), (3) reduce neuroinflammation, which is associated with neurodegeneration and neurotoxicity (He et al., 2016), and (4) enhance neuroprotective Parkin-mediated mitophagy (Moskal et al., 2020). Fasudil treatment resulted in significant improvement in motor symptoms in rodent models of PD (He et al., 2016; Tatenhorst et al., 2016), improved cognition in a transgenic mouse model of Alzheimer’s disease (Yan et al., 2021), and prolonged survival in a Parkin drosophila model (Moskal et al., 2020). Furthermore, Fasudil has shown dopaminergic effects in mouse models, suggesting that it could also have symptomatic effects in patients (Sortwell et al., 2013; Lopez-Lopez et al., 2020).

### 3.2 Rational for dose selection and trial design

Whereas in the licensed indication of Fasudil, the acute and vasoactive effect of the drug is sought for, and a three times daily regimen is required, treatment in chronic neurodegenerative disorders requires long-term application of the drug. In this clinical trial, the treatment period of 22 days followed by a wash-out phase of 28 days has been chosen to assess symptomatic effects and to establish a safety profile of an administration regime suitable for potential long-term treatment (Holloway et al., 2004). Oral application of Fasudil is expected to result in better treatment adherence long-term than intravenous application. Fluctuating patients are excluded from the clinical trial to not obscure symptomatic effects. An additional efficacy endpoint after 10 days will evaluate early symptomatic effects both in motor and nonmotor domains of PD in a subset of assessments. In this clinical trial, the licensed cumulative dosage of 3 × 30 mg/day (IV) for 14 days was transformed to 2 × 30 mg/day IV (corresponding to 2 × 44 mg/day *per os*) for 21 days, therefore adapting to a regime eligible for long-term treatment, while considering the approved cumulative dosage. This calculation is based on findings from a pharmacokinetic study, in which treatment with 45 mg Fasudil IV reached a mean peak level of 22.8 ng/mL Hydroxyfasudil, the active metabolite of Fasudil, in CSF (Hanada et al., 2005). Considering linear pharmacodynamics, peak levels after application of 15 or 30 mg Fasudil IV should be expected to be ~7.5 or 15 ng/mL, respectively, in the CSF. This corresponds well to the levels of Hydroxyfasudil, which were measured in mouse CSF (8.8 ng/mL) after oral treatment with Fasudil and resulted in beneficial effects on histological and behavioral parameters in multiple independent mouse models of PD (Tönges et al., 2012; Tatenhorst et al., 2016). To administer Fasudil as oral formulation, we performed an oral bioavailability trial (SAFE-ROCK, Eudra-CT-Nr.: 2019-001805-26) that showed excellent bioavailability of oral Fasudil compared to Fasudil IV. The area under the curve of Hydroxyfasudil concentrations was ~45% higher after the administration of the intravenous treatment compared to the oral treatment (Wolff et al., in revision). Therefore, in the present trial, two dosages adapted to this difference of 45% are used: 22 and 44 mg *per os* corresponding to 15 and 30 mg IV. Placebo was chosen as comparator because no gold-standard disease-modifying treatment for Parkinson’s disease exists to date. Because Fasudil has a slightly bitter taste, the placebo solution is supplemented with a bittering agent. Quinine dihydrochloride solution is frequently used in clinical trials to generate a bitter placebo solution. To this, 12.5 mg quinine dihydrochloride per placebo dosage are used, which is not pharmacologically active in this dosage.

### 3.3 Risk and benefit analysis

There is an ongoing need to develop disease modifying therapies for PD (Lang and Espay, 2018; McFarthing et al., 2023). Current treatment is limited to reduce symptoms but cannot prevent continuous symptomatic deterioration and progressing neuropathology. It is therefore an important aim to develop effective treatments that can stop or slow the course of the disease. Over the last decades, many therapeutics have demonstrated promising effects *in vitro* and animal models, but no effect could yet be translated into clinical disease-modifying effectiveness. Reasons for this are multifaceted and may relate to insufficient representation of disease pathology by available models, drug-related pharmacokinetic differences in humans, inadequately designed trials with too short follow-up periods as well as insufficient measures for long-term disease progression (Vijiaratnam et al., 2021). This makes development of disease modifying therapies time consuming and expensive. Therefore, drug repurposing – the evaluation of an already approved medication in a new disease or condition – can play a critical role in this development (Fletcher et al., 2021). Fasudil has been marketed for more than 25 years with a promising safety and tolerability profile (Suzuki et al., 2007). Most of its safety data originates from acute treatment over short time (14 days), yet Fasudil has been tested for longer treatment periods of up to 12 weeks in exploratory settings of vascular diseases (Vicari et al., 2005; Fukumoto et al., 2013). In this setting, the most common side effects were mild to moderate and did not occur significantly more frequent than in the placebo group. However, intracranial hemorrhage is listed as a side effect of treatment with Fasudil, which is most likely related to the indication of treatment instead of Fasudil itself. Bleeding relapse after intracerebral hemorrhage occurred in up to 30% within 1 year (Passero et al., 1995) and in 4% after an aneurysmal subarachnoid hemorrhage (Starke and Connolly, 2011), which is even more frequent than findings in Fasudil-treated patients where hemorrhages occurred in <2% (Suzuki et al., 2007). However, to exclude conditions that predispose for the occurrence of intracranial hemorrhages, patients with intracerebral aneurysms will be excluded from the current trial and those with a positive family history for aneurysms need to present an unremarkable cerebrovascular imaging result. The administration of placebo in our study population is acceptable since both placebo and Fasudil are given as add-on to the routine symptomatic therapy. On October 2nd, 2022, a search was performed in www.drugbank.ca (global drug properties) to identify known interactions between Fasudil and anti-parkinsonian drugs used for the treatment of early PD. No interaction was determined for Fasudil and levodopa/benserazide, levodopa/carbidopa, ropinirol, pramipexole, piribidil, entacapon, opicapone, amantadine, rasagiline, and selegiline. Fasudil is not listed on the Drug Interactions Flockhart Table™ of inhibitors or inducers of P450.^4^ The safety of the trial participants is of primary importance, and risks associated with the clinical trial participation have been weighed against the anticipated benefit for the clinical trial participants. All clinical data available so far provide reasonable evidence that Fasudil is eligible and safe for treatment in patients but data in PD patients are currently lacking. Based on preclinical findings, short-term symptomatic improvement seems possible. Although long-term benefits for the participants may be limited, they cannot be ruled out. Taken together, the benefits outweigh the risks.

Concluding, ROCK-PD is investigating safety, tolerability, and symptomatic efficacy of the ROCK-inhibitor Fasudil, which is a promising, potentially disease modifying molecule for PD. Based on the safety data and pharmacokinetic profile of Fasudil determined in this study, future clinical trials can be planned. Most importantly, this clinical trial can lay grounds for studies with a longer treatment duration to evaluate both symptomatic efficacy and disease modifying properties.

## 4 Ethics

This clinical trial will be conducted in accordance with the current ICH-GCP-guidelines and Declaration of Helsinki. Written informed consent will be obtained from all participants. Prior to study start the following documents were obtained:

- Approval of ethics committees (lead ethics committee at TU München [2022-443-Af-NP] and local ethics committees).
- Approval of national competent authority: Bundesamt für Arzneimittel und Medizinprodukte (BfArM).
- Notification to applicable regional authorities.
- Trial insurance.

Irrespective of the study outcome, the results will be disseminated through www.clinicaltrials.gov, in a peer-reviewed, international journal, and national and international conferences respecting the privacy of the participants. A lay summary of the results will be available on the study website.

## Ethics statement

The studies involving humans were approved by ethics committee at TU München [2022-443-Af-NP]. The studies were conducted in accordance with the local legislation and institutional requirements. The participants provided their written informed consent to participate in this study.

## Author contributions

AW: Conceptualization, Methodology, Project administration, Writing – original draft. HB: Conceptualization, Methodology, Project administration, Writing – review & editing. YR: Investigation, Methodology, Writing – review & editing. JZ: Investigation, Methodology, Writing – review & editing. DA: Conceptualization, Methodology, Writing – review & editing. OR: Conceptualization, Methodology, Writing – review & editing. RW: Conceptualization, Methodology, Writing – review & editing. AH: Conceptualization, Formal analysis, Methodology, Software, Writing – review & editing. PL: Conceptualization, Funding acquisition, Methodology, Supervision, Writing – review & editing.

## Footnotes

^1^ www.rock-als.uni-goettingen.de

^2^ https://www.kompetenznetz-parkinson.de/klinische-zentren

^3^ https://rock-pd.med.tum.de/en

^4^ http://medicine.iupui.edu/clinpharm/ddis/index
